# Living in the United States Stroke Belt and cognitive change in the Women’s Health Study: a cohort study

**DOI:** 10.1186/s12883-026-05224-6

**Published:** 2026-08-17

**Authors:** Alice C. von Alvensleben, Julie E. Buring, Ricarda S. Schulz, Paula J. Filser, Jae H. Kang, Pamela M. Rist, Tobias Kurth

**Affiliations:** 1 Institute of Public Health, Charité – University Medical Center Berlin, corporate member of Freie Universität Berlin and Humboldt-Universität zu Berlin, Berlin, 10117 Germany; 2 Division of Preventive Medicine, Department of Medicine, Brigham and Women’s Hospital, Harvard Medical School, Boston, MA USA; 3 Department of Health Care Management, Technische Universität Berlin, Berlin, Germany; 4 Channing Division of Network Medicine, Department of Medicine, Brigham and Women’s Hospital, Harvard Medical School, MA Boston, USA

**Keywords:** Cognition, Cognitive change, Cognitive decline, Stroke Belt, Women’s health, Cohort study, Epidemiology

## Abstract

**Background and objectives:**

Residence in the United States Stroke Belt has been linked to a higher stroke mortality rate, raising the question of whether this increased risk is also associated with other brain health outcomes, such as cognitive change, defined as within-person changes in cognitive performance over time. This study aimed to investigate if residing in the Stroke Belt is associated with cognitive decline compared to residing outside this region in a large cohort of women.

**Methods:**

We used data from the cognitive cohort of the Women’s Health Study, a U.S.-based cohort of predominantly White women currently or formerly employed in health professions. Our analyses included 6,362 women aged ≥65 years at baseline who underwent cognitive assessments. The exposure was residency in the Stroke Belt. Cognitive change was assessed as the primary outcome using repeated measures of a global cognitive composite score. Linear mixed-effects models were applied, adjusted for age, education, and lifestyle factors, measured by Life’s Simple 7 score with random effects for each of the participating women (fully adjusted model). Secondary analyses used logistic regression to examine the association between Stroke Belt residency and the odds of belonging to the first quintile or first decile of the cognitive change distribution (secondary outcome), representing women with the greatest cognitive decline.

**Results:**

Of the participating women, 1,131 (17.8%) resided within the Stroke Belt. Compared to women residing outside the Stroke Belt, women residing in the Stroke Belt did not have significantly different rates of cognitive decline. The fully adjusted difference in change in the global cognitive composite score from the first to the last assessment between women living inside and outside the Stroke Belt was β = -0.03, SE = 0.02, *p* = 0.233. Women residing in the Stroke Belt had higher odds of being in the first quintile (adjusted odds ratio (OR) 1.20, 95% confidence interval (CI) 1.00–1.42) and in the first decile (adjusted OR 1.43, 95% CI 1.14–1.78) of the cognitive change distribution.

**Discussion:**

Residence in the U.S. Stroke Belt was not associated with overall differences in cognitive decline trajectories, although secondary analyses suggested a possible increase in more pronounced decline among women living in the region.

**Trial Registration Information:**

ClinicalTrials.gov Identifier: NCT00000479; retrospectively submitted: October 27, 1999.

**Supplementary Information:**

The online version contains supplementary material available at https://doi.org/10.1186/s12883-026-05224-6.

## Introduction

The Stroke Belt is a region in the Southeast of the United States (U.S.), encompassing Alabama, Arkansas, Georgia, Indiana, Kentucky, Louisiana, Mississippi, North Carolina, South Carolina, Tennessee, and Virginia [1]. Since 1940, this region has consistently exhibited stroke mortality rates that are 10% higher than those in the rest of the U.S., underscoring significant regional disparities in stroke outcomes [2, 3]. Studies have shown that factors contributing to stroke, such as lifestyle factors [4], are also linked to an elevated risk of cognitive decline [5–7], raising the question of whether living in the Stroke Belt, where these factors are more prevalent, is linked to an elevated risk of cognitive decline.

Cognitive change is defined as within-person changes in cognitive performance over time [8]. Cognitive decline encompasses both non-pathological cognitive aging and pathological forms, with the former involving reductions in mental abilities such as memory and executive functions beginning in early adulthood and being influenced by genetics, health, diet, and medical conditions [9, 10]. With an aging population, both forms of cognitive decline are expected to become more prevalent, posing significant societal and economic challenges and highlighting the need for effective prevention strategies [11].

The Stroke Belt has received considerable attention due to its potential association with various health outcomes [1]. While stroke incidence and mortality have been extensively studied in this region [12–16], the relationship between residing in the Stroke Belt and cognitive change remains understudied. Previous studies, such as the REGARDS study, focused on cognitive impairment and included limited adjustment for confounding, and the STAR study focused on late-life cognition [13, 17].

This study addresses, therefore, a research gap by examining a different population, consisting of a large cohort of predominantly White women who had follow-up data to conceptualize cognitive change. Given that women experience a greater lifetime burden of stroke, including a higher lifetime risk of stroke, greater stroke-related mortality, and poorer functional outcomes after stroke [18, 19], and may also be at increased risk of dementia [20] and more rapid progression from mild cognitive impairment to dementia than in men [21], investigating cognitive change in this population may provide important insights into regional disparities in cognitive aging.

We therefore aimed to investigate if residing in the Stroke Belt is associated with cognitive decline compared to residing outside this region using data originating from the U.S.-based Women’s Health Study.

## Methods

### Study population

The Women’s Health Study (WHS) (ClinicalTrials.gov Identifier: NCT00000479; retrospectively submitted: October 27, 1999) began as a randomized, double-blinded, placebo-controlled trial which enrolled participants in 1992–1995. It was designed to test the effects of low-dose aspirin and vitamin E on preventing cardiovascular diseases (CVD) and cancer among 39,876 mostly white (>95%) female health professionals in the U.S. aged 45 years and older at baseline [22]. Women had to be free of CVD, cancer (except nonmelanoma skin cancer), and other major diseases at baseline of the WHS [22].

The main trial concluded in March 2004, transitioning into an observational cohort study with ongoing annual follow-up. The study design and its results have been previously reported [22, 23].

All women provided written informed consent. The study was approved by the Institutional Review Board of Brigham and Women’s Hospital, Boston, Massachusetts (IRB number observational follow-up: 2004P001661 and cognitive cohort: 1999P002903).

In 1998, a cognitive cohort commenced among women aged 65 years or older who were still active in the WHS. Of the 7,175 eligible women, 6,377 (88.9%) completed the initial cognitive assessment by telephone 5.6 years (mean) post-randomization (see Supplementary Fig. 1) [22]. We excluded women who, at baseline of the WHS, resided outside the U.S. Women underwent two additional follow-up assessments at approximately two-year intervals [22]. Secondary analyses were restricted to participants who completed the subtests required to calculate the global cognitive score at the first and last cognitive assessments. Information on inclusion into the study is presented in Supplementary Fig. 1.

High follow-up was maintained throughout, with rates nearly identical between the treatment and placebo groups [22].

### Exposure assessment

The primary exposure of interest was residence in the U.S. Stroke Belt, defined as residing in one of the following states at the time of enrollment into the WHS: Alabama, Arkansas, Georgia, Indiana, Kentucky, Louisiana, Mississippi, North Carolina, South Carolina, Tennessee, and Virginia [1].

### Outcome assessment

Three cognitive assessments were performed, starting with the baseline evaluation in 1998 and followed by two follow-up assessments at approximately two-year intervals in 2000 and 2002 [23]. To assess cognitive status, a comprehensive telephone-based cognitive test battery was administered by trained nurses. The battery included five tests (I-V) designed to evaluate general cognition, verbal memory, and executive functioning [22]:

(I) To assess general cognition, the Telephone Interview for Cognitive Status (TICS) was used, which is a validated telephone adaptation of the Mini-Mental State Examination [24, 25]. The TICS measures global cognitive function (score range 0 – 41) with items covering orientation, immediate and delayed verbal recall, serial subtraction, and other cognitive skills [26].
(II + III) Verbal memory was assessed using the immediate and delayed recall trials of the East Boston Memory Test [27], where women were asked to recall 12 key elements of a short paragraph both immediately and after a 15-minute delay (score range 0 – 12).
(IV)  Additionally, delayed verbal memory was further evaluated using a delayed recall of a 10-word list from the TICS (score range 0 – 10).
(V)  Category fluency, such as in language and executive function, was assessed through a task, where women were asked to name as many animals as possible within one minute [28]

Our primary outcome was global cognitive function, which was calculated as follows: raw test scores from each of the cognitive tests were standardized using the means and standard deviations from the first assessment to generate *z*-scores as previously applied in the context of the WHS [22]. For the two follow-up assessments, cognitive test scores were standardized using the same baseline means and standard deviations, ensuring that all scores were expressed on a common scale relative to baseline performance. At each assessment, the global cognitive composite score was calculated by averaging the standardized cognitive test scores for each woman.

For secondary analyses, cognitive change over time was additionally quantified by calculating the differences between the global composite *z*-score at the last and that at the first cognitive assessment. To characterize substantial cognitive decline, threshold-based definitions consistent with previous studies in the context of the WHS were applied [23, 29]. Women in the first quintile and first decile of the resulting cognitive change distribution were classified as experiencing pronounced cognitive decline. Dichotomous indicator variables were created to indicate whether women belonged to these groups.

### Covariate assessment

To assess lifestyle factors, we utilized the American Heart Association Life’s Simple 7 score [30], which comprises four behavioral factors: smoking, diet, physical activity, and Body-Mass-Index (BMI), and three biomarkers: fasting glucose, blood cholesterol, and blood pressure. Given the absence of fasting blood glucose measurements at baseline for all WHS participants, we used self-reported diabetes or HbA1c levels >5% as a proxy. Similarly, self-reports of high cholesterol and total cholesterol levels were used for women who did not provide a blood sample at baseline which could be assayed for total cholesterol. Each participant received one point for meeting the following criteria based on AHA guidelines [23]: Non-smoker, BMI between 18.5 and 24.9 kg/m^2^, untreated total cholesterol <200 mg/dL, untreated resting blood pressure <120/<80 mmHg, HbA1c <5% or no history of diabetes, adequate physical activity (≥150 min/week of moderate intensity or ≥75 min/week of vigorous intensity), and a healthy diet (defined as meeting at least two of the following: ≥4.5 cups/day of fruits and vegetables, ≥2 servings/week of fish and ≥3 servings/day of fiber-rich whole grains, <1,500 mg/day of sodium, and <36 oz/week of sugar-sweetened beverages). We then classified participants into three categories based on their score: Poor (0–3), Intermediate (4–5), and Optimal (6–7). Similar approaches have been applied and described in previous studies [30, 31].

Age was assessed as continuous in years and education in six categories (1) Licensed Practical Nurse or Licensed Vocational Nurse training, (2) 2-year Associate’s degree-Registered Nurse, (3) Diploma program (3-year-Registered Nurse), (4) Bachelor’s degree or Bachelor of Science in nursing, (5) Master’s degree, (6) Doctoral degree (including Doctor of Medicine).

### Statistical analysis

#### Descriptive analysis

Mean values together with standard deviations were reported for normally distributed continuous variables. Medians (with minimum and maximum values) were presented for non-normally distributed continuous variables and proportions were provided for categorical data. We compared baseline characteristics and test scores at each time point by residency in the Stroke Belt.

#### Primary analysis

Linear mixed-effects models were employed for the primary analysis. As in a previous WHS study [28], we modeled time nominally instead of linearly, given the lack of a linear relationship between time and cognitive performance. The initial model included the interaction between cognitive assessments and Stroke Belt residency to account for joint effects of Stroke Belt residency and time, and incorporated random intercepts for individual women to account for within-subject correlation and baseline differences.

A second model further adjusted for age and education, as these key demographic factors could influence cognitive outcomes [32]. A third model additionally accounted for LS7 categories to account for the potential impact of lifestyle and health factors on the association between Stroke Belt residency and cognitive decline. The reference categories for all models were residing outside the Stroke Belt and the first cognitive assessment.

The primary contrast of interest was the difference in the change in the global cognitive composite score from the first to the last cognitive assessment among women residing inside vs. outside the Stroke Belt, estimated by the interaction between Stroke Belt residence and the last cognitive assessment.

#### Secondary analyses

A priori we specified secondary analyses in which we examined the association between Stroke Belt residence and experiencing large changes in cognitive function, similar to prior work in WHS [23, 29]. Therefore, we used logistic regression to investigate the association between residing in the Stroke Belt and belonging to the first quintile of the cognitive change distribution. This quintile represents the participants who experienced the greatest cognitive decline during follow-up. Thus, the outcome was defined as belonging to the group of participants with the greatest cognitive decline relative to the study population. We further assessed the association between residing in the Stroke Belt and belonging to the first decile of the cognitive change distribution to explore the robustness of our findings. The reference category for all analyses was residing outside the Stroke Belt, and we used the same adjustment sets as in the primary analyses.

### Handling of missing data

Education (n = 94, 1.5%) and LS7 score (*n* = 549, 8.6%) were the only variables in the adjustment set with missing data. We handled missingness by assigning participants with missing education to the most common category “*(3) Diploma program (3-year–Registered Nurse)*” and missing LS7 values to “*Intermediate*”, the most frequent category. This approach allowed us to retain the full study population while minimizing the potential impact of missing data on the study findings [32].

To assess potential selection and survival bias, we compared baseline characteristics for those who completed all three assessments versus those who only completed the first assessment.

### Effect modification

We evaluated whether the association between residing in the Stroke Belt and change in the global cognitive composite score (our primary outcome) was modified by age (above vs. at or below baseline median age) and education was dichotomized (bachelor’s degree or higher vs. lower education). Effect modification was assessed by including three-way interaction terms between Stroke Belt residency, cognitive assessment, and the respective effect modifier. Because the primary contrast of interest was the difference in change from the first to the last cognitive assessment, we focused on the three-way interaction term involving the last cognitive assessment. The model evaluating effect modification also adjusted for age (continuous) and education in 6 categories.

For all statistical analyses, we used a two-sided significance level of α = 0.05. All statistical analyses were conducted using *R* version 4.5.2 [33], and RStudio, version 2024.09.1 + 394.

## Results

After excluding 15 women who, at the time of baseline cognitive assessment, resided outside the U.S., we included 6,362 women in our primary analyses.

For the secondary analyses, we only considered women who completed the subtests required to calculate the global cognitive score at the first and last cognitive assessment (*n* = 5,206).

### Descriptive analysis

Among the 6,362 women included in the study, 1,131 (17.8%) resided inside the Stroke Belt, while 5,231 (82.2%) lived outside this region. The mean age of the women was 66.2 years (SD 4.1) at baseline, and 95.6% identified as non-Hispanic White.

Table 1 provides a detailed overview of the baseline characteristics of women categorized by their residence within or outside of the Stroke Belt. Women living inside the Stroke Belt exhibited slightly lower educational attainment compared to those residing outside the Stroke Belt. Specifically, 71.5% of women in the Stroke Belt had less than a bachelor’s degree, compared to 65.0% of women outside the Stroke Belt. The prevalence of overweight or obesity was similar for women living in the Stroke Belt (50.6% having a BMI above 24.9 kg/m²) and for those living outside the region (51.3%). Smoking rates were marginally higher in women residing within the Stroke Belt, with 11.2% of women being current smokers compared to 9.7% outside the region.

**Table 1:** Baseline characteristics of included women according to Stroke Belt residence status in the Women’s Health Study (*N* = 6,362)

	Living outside the Stroke Belt (*n* = 5,231)	Living inside the Stroke Belt (*n* = 1,131)	Overall (*N* = 6,362)
Age (years)			
Mean (SD)	66.3 (4.08)	66.1 (4.05)	66.2 (4.08)
Median [Min, Max]	65.5 [60.4, 85.0]	65.4 [60.4, 89.9]	65.5 [60.4, 89.9]
Race/Ethnicity, n (%)			
Non-Hispanic White	5002 (95.6%)	1079 (95.4%)	6081 (95.6%)
Hispanic	40 (0.8%)	0 (0%)	40 (0.6%)
African American/Black	91 (1.7%)	33 (2.9%)	124 (1.9%)
Asian or Pacific Islander	40 (0.8%)	9 (0.8%)	49 (0.8%)
American Indian or Alaskan Native	8 (0.2%)	0 (0%)	8 (0.1%)
Missing or Unknown	50 (1.0%)	10 (0.9%)	60 (0.9%)
Education, n (%)			
LPVN, associate’s degree, registered nurse	3401 (65.0%)	809 (71.5%)	4210 (66.2%)
Bachelor’s degree, Master’s degree, Doctorate	1749 (33.4%)	309 (27.3%)	2058 (32.3%)
Missing	81 (1.5%)	13 (1.1%)	94 (1.5%)
Body mass index, n (%)			
Below 18.5 kg/m²	64 (1.2%)	16 (1.4%)	80 (1.3%)
18.5–24.9 kg/m²	2479 (47.4%)	542 (47.9%)	3021 (47.5%)
Above 24.9 kg/m²	2684 (51.3%)	572 (50.6%)	3256 (51.2%)
Missing	4 (0.1%)	1 (0.1%)	5 (0.1%)
Smoking status, n (%)			
Never	2715 (51.9%)	618 (54.6%)	3333 (52.4%)
Past	2004 (38.3%)	386 (34.1%)	2390 (37.6%)
Current	506 (9.7%)	127 (11.2%)	633 (9.9%)
Missing	6 (0.1%)	0 (0%)	6 (0.1%)
Physical activity, n (%)			
Rarely/never	2223 (42.5%)	518 (45.8%)	2741 (43.1%)
< 1 time/week	861 (16.5%)	178 (15.7%)	1039 (16.3%)
2–3 times/week	1488 (28.4%)	331 (29.3%)	1819 (28.6%)
>=4 times/week	657 (12.6%)	101 (8.9%)	758 (11.9%)
Missing	2 (0.0%)	3 (0.3%)	5 (0.1%)
Blood Pressure, n (%)			
<140/90 mm Hg	3116 (59.6%)	697 (61.6%)	3813 (59.9%)
>=140/90 mm Hg	2114 (40.4%)	433 (38.3%)	2547 (40.0%)
Missing	1 (0.0%)	1 (0.1%)	2 (0.0%)
Healthy Diet*, n (%)			
Healthy	731 (14.0%)	185 (16.4%)	916 (14.4%)
Not healthy	4314 (82.5%)	905 (80.0%)	5219 (82.0%)
Missing	186 (3.6%)	41 (3.6%)	227 (3.6%)
History of Diabetes, n (%)			
no	5053 (96.6%)	1085 (95.9%)	6138 (96.5%)
yes	177 (3.4%)	46 (4.1%)	223 (3.5%)
Missing	1 (0.0%)	0 (0%)	1 (0.0%)
Total Cholesterol, n (%)			
<200 mg/dL	1413 (27.0%)	322 (28.5%)	1735 (27.3%)
>200 mg/dL	3586 (68.6%)	761 (67.3%)	4347 (68.3%)
Missing	232 (4.4%)	48 (4.2%)	280 (4.4%)
Life’s Simple 7 Score, n (%)			
Poor (0–3)	1573 (30.1%)	373 (33.0%)	1946 (30.6%)
Intermediate (4–5)	2640 (50.5%)	553 (48.9%)	3193 (50.2%)
Optimal (6–7)	565 (10.8%)	109 (9.6%)	674 (10.6%)
Missing	453 (8.7%)	96 (8.5%)	549 (8.6%)

*Min*  Minimum, Max Maximum,* SD *Standard Deviation

*Healthy diet was defined as meeting at least two of the following: ≥4.5 cups/day of fruits and vegetables, ≥2 servings/week of fish, ≥3 servings/day of fiber-rich whole grains, <1500 mg/day of sodium, and <36 oz/week of sugar-sweetened beverages

Levels of physical activity were lower among women in the Stroke Belt, with fewer engaging in exercise four or more times per week. Also, women living inside the Stroke Belt had a slightly higher proportion in the “Poor” category of LS7 score (33.0% versus 30.1%) compared to women living outside the Stroke Belt.

In Supplementary Table 1, we provide information about women who completed all three assessments as well as about women who did not complete assessments two or three. Women who missed the second or last cognitive assessments were more likely to be current smokers, to have a history of diabetes and high blood pressure. Upon comparing the distribution of participants across the LS7 categories, both for those who completed and those who did not participate in each assessment, we found minimal deviation of up to 3.4%. This suggests that the influence of the LS7 categories on participation in the last cognitive assessment was presumably small. Performance in each individual cognitive test at each assessment is summarized in Supplementary Table 2.

### Primary analysis

As shown in Table 2, we observed that all of the interaction terms between Stroke Belt residence and time were near the null and did not reach statistical significance, suggesting that there is no evidence for greater decline rates in cognition for women living inside the Stroke Belt in all models (global cognition: β = -0.03, SE = 0.02, *p* = 0.233).

**Table 2:** Associations between Stroke Belt residence and cognitive composite score over time in the Women’s Health Study (*N* = 6,362)

Residence status and cognitive assessment	Global Cognitive Score	
	Coefficient (SE)	*P* value
Model^1^		
Second assessment	0.07 (0.01)	<0.001
Last assessment	0.03 (0.01)	<0.001
Living inside the Stroke Belt	-0.07 (0.02)	0.003
Stroke Belt*Second assessment	0.02 (0.02)	0.43
Stroke Belt*Last assessment	-0.03 (0.02)	0.21
Model^2^		
Second assessment	0.07 (0.01)	<0.001
Last assessment	0.03 (0.01)	<0.001
Living inside the Stroke Belt	-0.06 (0.02)	0.043
Stroke Belt*Second assessment	0.02 (0.02)	0.40
Stroke Belt*Last assessment	-0.03 (0.02)	0.23
Model^3^		
Second assessment	0.07 (0.01)	<0.001
Last assessment	0.03 (0.01)	<0.001
Living inside the Stroke Belt	-0.06 (0.02)	0.005
Stroke Belt*Second assessment	0.02 (0.02)	0.41
Stroke Belt*Last assessment	-0.03 (0.02)	0.23

*SE*  standard error

Coefficients were estimated using linear mixed-effects models with random intercepts for individual participants

Reference categories were residence outside the Stroke Belt and the first cognitive assessment

The coefficient for living inside the Stroke Belt represents the difference in global cognitive composite score at the first assessment. Interaction terms estimate the additional change from the first to the respective follow-up assessment among women living inside versus outside the Stroke Belt

Model 1 unadjusted (interaction between Stroke Belt and time)

Model 2 additionally adjusted for age and education

Model 3 additionally adjusted for age, education, and Life’s Simple 7

**Fig. 1:**
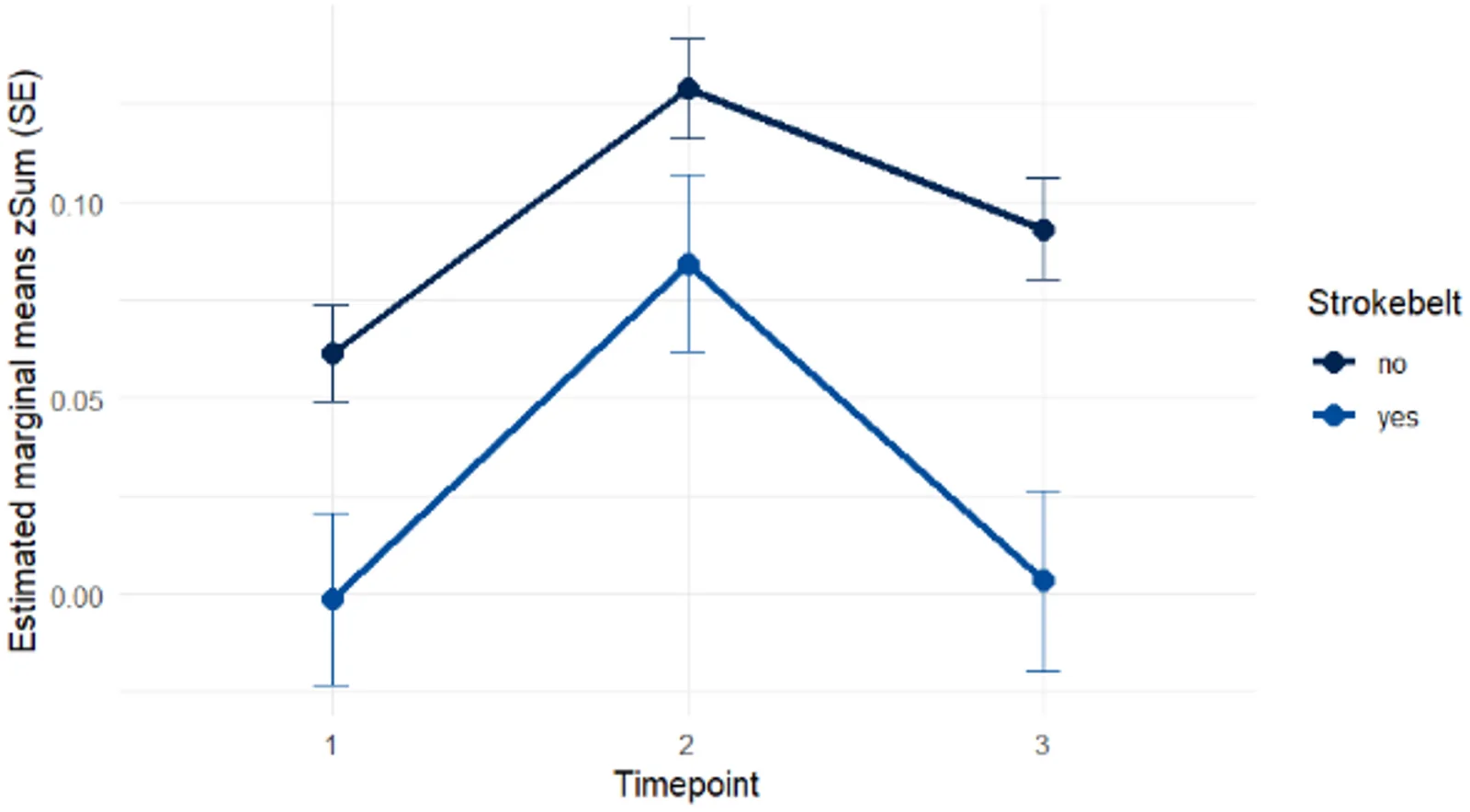
Mean trajectories of global cognitive composite scores across three cognitive assessments according to Stroke Belt residency. Estimated marginal means (SE) of the global cognitive composite z-score across the three cognitive assessments by Stroke Belt residency. Estimates are derived from models adjusted for age, education, and Life’s Simple 7 score. Error bars represent standard errors. Cognitive change over time was assessed using linear mixed-effects models

Women living inside the Stroke Belt initially had a significantly lower global cognitive composite score in comparison to women living outside the Stroke Belt in all models (global cognition: β = −0.06, SE = 0.02, p = 0.005) in the fully adjusted model (Model 3). The results also indicate that women living both in- and outside the Stroke Belt have a slightly higher global cognitive composite score during the second assessment (β = 0.07, SE = 0.01, *p* <0.001) and last assessment (β = 0.03, SE = 0.01, *p* <0.001) than the first assessment (Fig. 1).

### Secondary analyses

In the unadjusted analysis, individuals living inside the Stroke Belt had an OR of 1.18 (95% CI: 0.99–1.40) for belonging to the first quintile of the cognitive change distribution, suggesting a statistically non-significant increase in the odds of cognitive decline among Stroke Belt residents. Adjusting for age and education resulted in statistically significant increased odds of 1.20 (95% CI: 1.00–1.42). Further adjusting for LS7 resulted in a similar increase in odds of cognitive decline with an odds ratio of 1.20 (95% CI: 1.00–1.42) (Table 3).

**Table 3:** Odds Ratios for being in the first quintile of the cognitive change distribution according to Stroke Belt residency in the Women’s Health Study (*N* = 5,218)

Cognitive Change	≤ 20% (*n* = 1,041) OR (95% CIs)	> 20% (*n* = 4,165) OR (ref.)
Living inside the Stroke Belt (*n* = 915)		
unadjusted	1.18 (0.99–1.40)	1 (ref.)
adjusted^1^	1.20 (1.00–1.42)	1 (ref.)
adjusted^2^	1.20 (1.00–1.42)	1 (ref.)

*CI* Confidence Interval,* OR* Odds Ratio

^1^Adjusted for age and education

^2^Adjusted for age, education and Life’s Simple 7

In the unadjusted analysis examining the association between residing in the Stroke Belt and being in the first decile of the cognitive change distribution, individuals residing inside the Stroke Belt had a statistically significantly higher OR of 1.41 (95% CI: 1.13–1.75) for belonging to the first decile of the cognitive change distribution compared to those living outside the Stroke Belt.

After including age and education to the model, the OR increased slightly to 1.43 (95% CI: 1.14–1.78).

Further adjustment for the LS7 score did not change this estimate, suggesting that the Stroke Belt residents are at a higher risk of belonging to the first decile of the cognitive change distribution, independent of their lifestyle and health behaviors as measured by the LS7 score, age, and education (Table 4).

**Table 4:** Odds Ratios for being in the first decile of the cognitive change distribution according to Stroke Belt residency in the Women’s Health Study (*N* = 5,206)

Cognitive Change	≤ 10% (*n* = 521) OR (95% CIs)	> 10% (*n* = 4,685) OR (reference)
Living inside the Stroke Belt (*n* = 915)		
unadjusted	1.41 (1.13–1.75)	1 (ref.)
adjusted^1^	1.43 (1.14–1.78)	1 (ref.)
adjusted^2^	1.43 (1.14–1.78)	1 (ref.)

CI Confidence Interval, OR Odds Ratio

ref. reference group (women residing outside the Stroke Belt)

^1^Adjusted for age and education

^2^Adjusted for age, education and Life’s Simple 7

### Effect modification

We did not observe statistically significant effect modification by age for the association between Stroke Belt residency and change in global cognitive composite score from the first to the second or last cognitive assessment (*p* = 0.70). In contrast, we found statistically significant effect modification by education. At the last assessment, there was a statistically significant effect modification by education (*p* = 0.046). Women residing in the Stroke Belt who were in the lower education category showed greater cognitive decline (β = −0.06, SE = 0.03, *p = *0.039), whereas women residing in the Stroke Belt with higher education showed no significant change in cognitive function (β = 0.04, SE = 0.04, *p = *0.33).

## Discussion

### Key results

In this large cohort of women, we investigated whether residence in the U.S. Stroke Belt was associated with change in global cognitive function. Overall, we did not observe meaningful differences in change in the global cognitive composite score from the first to the last assessment between women living inside and outside the Stroke Belt. Although the primary analyses did not suggest differences in overall decline trajectories, in secondary analyses using categorical definitions of decline, women residing in the Stroke Belt showed higher odds of more pronounced cognitive decline.

Associations remained after adjustment for LS7, indicating that factors beyond those captured by LS7 may contribute to cognitive decline among women residing in the Stroke Belt.

We observed evidence of effect modification by education at the last assessment, although the differences between binary education groups were small. Women with lower education showed a greater decline, whereas those with higher education showed no significant difference in cognitive performance. Given the modest magnitude and absence of similar findings in earlier assessments, this result should be interpreted with caution.

### Comparison with other studies

Our findings on cognitive decline among women in the Stroke Belt align with previous research and offer some additional insights. A study that investigated incident cognitive impairment in the Stroke Belt using the Reasons for Geographic and Racial Differences in Stroke (REGARDS) cohort reported that residents of the Stroke Belt had a higher adjusted odds ratio (OR = 1.18; 95% CI: 1.07–1.30) of incident cognitive impairment than non-Stroke Belt residents [13, 14]. This is consistent with our findings, which also indicate a higher risk of cognitive decline among women residing in the Stroke Belt, particularly evident in the first decile of the cognitive change distribution.

Similarly, a more recent study from 2020, which explored the impact of the Stroke Belt birth state on late-life cognition in African Americans, found that those born in the Stroke Belt exhibited worse cognitive outcomes in terms of executive function and semantic memory [17]. Although their focus was on birth state rather than current residence, their results support that geographic factors associated with the Stroke Belt are linked to poorer cognitive health outcomes. Our findings, showing elevated odds ratios of cognitive decline among U.S. residents in the Stroke Belt, complement these observations by highlighting that residing in the Stroke Belt in adulthood is associated with higher odds of cognitive decline [17]. However, we could not explore whether this association is independent of birth state or residing in the Stroke Belt during other time periods.

### Strengths and limitations

The strengths of this study include the large sample size and available data on lifestyle and health factors. The homogeneity of the study population may also enhance comparability by reducing variability in factors such as healthcare access.

However, several limitations should be acknowledged when interpreting the results. We used the predefined Stroke Belt classification available within the Women’s Health Study, which differs slightly from the definitions applied in studies such as REGARDS and STAR by including a broader set of states. These differences in exposure classification may have attenuated regional contrasts and could partly explain differences between our findings and those reported previously. In addition, residence was assessed only at enrollment in the WHS, and information on participants’ birthplaces, duration of residence within or outside the Stroke Belt, and residential mobility over time was unavailable. Consequently, our study may not fully capture long-term geographic exposure, which has been suggested to influence the risk of cognitive impairment and dementia [34–36].

Additionally, reliance on self-reported data for LS7 covariates introduces the risk of misclassification. Although self-reports are a common method for collecting such data, they can be subject to errors and biases, which may impact the accuracy of the observed associations. Furthermore, the use of the LS7 score represents a limitation, as it does not include some conditions with established relevance for cognitive function, including a history of atrial fibrillation, chronic heart failure, coronary artery disease, and chronic kidney disease, among others. However, at the time of enrollment, women were unlikely to have these conditions, as the WHS specifically included participants who were free of cardiovascular disease and other major health conditions.

The study cohort consisted of a homogeneous group of middle-aged, predominantly white, health-conscious women working in the healthcare sector. This occupational group might possess different health knowledge and engage in healthier lifestyle practices than the general population. Consequently, the study population may not represent the broader population, limiting the generalizability of the findings to other demographic groups, particularly men, people of different ethnicities, and individuals with varying socioeconomic statuses. Furthermore, we observed little cognitive decline among participants, likely due to the relatively young age of the cohort, a presumably high cognitive reserve, and the short follow-up period. It is possible that the decline was not substantial enough to detect significant changes. While the study had quite a large sample size, the overall low incidence of cognitive decline may have limited the ability to detect smaller differences in risk between groups. In addition, repeated cognitive assessments conducted at two-year intervals may have introduced learning effects that attenuated measurable decline. Given the telephone-based nature of the cognitive assessments, we cannot exclude the possibility that some participants used strategies such as note-taking during testing, which may have contributed to improvements in cognitive scores over time. However, for this to explain our findings, such behaviors, and any resulting practice effects, would have had to differ systematically according to geographic region, for which we have no evidence. Comparisons of baseline characteristics (Supplementary Table 1) did not indicate substantial differences between women who completed all assessments and those who did not.

## Conclusion

In this large cohort of women, residence in the U.S. Stroke Belt was not associated with differences in overall cognitive decline trajectories. Secondary analyses suggested higher odds of more pronounced decline among women living in the Stroke Belt, indicating that specific aspects of cognitive change may warrant further investigation. Exploratory analyses also suggested possible effect modification by education, although the observed differences were modest. Future studies should further investigate the role of education, including potential pathways such as cognitive reserve, socioeconomic conditions, access to health care, and health-related behaviors. The mechanisms underlying these regional differences remain incompletely understood, and further research is warranted to better understand the contribution of possible factors to the observed regional differences in cognitive performance and to assess the generalizability of these findings to other populations, including men. 

## Supplementary Information


Supplementary Material 1.


## Data Availability

The data contains information that could compromise the privacy and consent of research participants, and it is, therefore, not publicly available.

## Abbreviations

BMI: Body Mass Index
CI: Confidence Interval
CVD: Cardiovascular Disease
HbA1c: Hemoglobin A1c
IRB number: Institutional Review Board Number
kg: Kilogram
LS7: Life’s Simple 7
m²: Square Meter
max: Maximum
min: Minimum
mmHg: Millimeters of Mercury
OR: Odds Ratio
REGARDS study: REasons for Geographic And Racial Differences in Stroke Study
SD: Standard Deviation
SE: Standard Error
STAR study: Study of Healthy Aging in African Americans
TICS: Telephone Interview for Cognitive Status
U.S: United States
WHS: Women’s Health Study
