# Supplementary material for: Living in the United States Stroke Belt and cognitive change in the Women’s Health Study: a cohort study
Source: BMC Neurol. 2026 Aug 17;26:555. doi: 10.1186/s12883-026-05224-6 (PMC13527909; doi:10.1186/s12883-026-05224-6)
Supplement: Supplementary file 1 [file 12883_2026_5224_MOESM1_ESM.docx]

**Supplementary Material**

**List of supplementary tables and figures**

**Supplementary Figure 1:** Flow Chart in participation in the cognitive cohort and cognitive assessments in the Women’s Health Study

**Supplementary Table 1**: Baseline characteristics of women with missing second or last cognitive assessment within the Women’s Health Study’s cognitive cohort (*N* = 6,362)

**Supplementary Table 2:** Cognitive test performance at each cognitive assessment by Stroke Belt residence at baseline (*N* = 6,362)

**Supplementary Figure 1:** Flow Chart in participation in the cognitive cohort and cognitive assessments in the Women’s Health Study

**Supplementary Table 1:** Baseline characteristics of women with missing second or last cognitive assessment within the Women’s Health Study’s cognitive cohort (*N* = 6,362)

|  |  | **Completed all assessments**  (*n* = 5,067) | **Missing second or last assessment**  (*n* = 1,295) | **Overall**  (*N* = 6,362) |
| --- | --- | --- | --- | --- |
|  | Age (years) |  |  |  |
|  | Mean (SD) | 66.1 (3.97) | 66.7 (4.45) | 66.2 (4.08) |
|  | Median [Min, Max] | 65.4 [60.4, 87.1] | 65.9 [60.4, 89.9] | 65.5 [60.4, 89.9] |
| Race/Ethnicity, n (%) | |  |  |  |
| Non-Hispanic White | | 4864 (96.0%) | 1217 (94.0%) | 6081 (95.6%) |
| Hispanic | | 28 (0.6%) | 12 (0.9%) | 40 (0.6%) |
| African American/Black | | 92 (1.8%) | 32 (2.5%) | 124 (1.9%) |
| Asian or Pacific Islander | | 36 (0.7%) | 13 (1.0%) | 49 (0.8%) |
| American Indian or Alaskan Native | | 6 (0.1%) | 2 (0.2%) | 8 (0.1%) |
| Missing or Unknown | | 41 (0.8%) | 19 (1.5%) | 60 (0.9%) |
|  | Education, n (%) |  |  |  |
|  | LPVN, associate’s degree, registered nurse | 3,315 (65.4%) | 895 (69.1%) | 4,210 (66.2%) |
|  | Bachelor’s degree, Master’s degree, Doctorate | 1671 (33.0%) | 387 (29.9%) | 2,058 (32.3%) |
|  | Missing | 81 (1.6%) | 13 (1.0%) | 94 (1.5%) |
|  | Body mass index, n (%) |  |  |  |
|  | Below 18.5 kg/m² | 56 (1.1%) | 24 (1.9%) | 80 (1.3%) |
|  | 18.5-24.9 kg/m² | 2,368 (46.7%) | 653 (50.4%) | 3,021 (47.5%) |
|  | Above 24.9 kg/m² | 2,642 (52.1%) | 614 (47.4%) | 3,256 (51.2%) |
|  | Missing | 1 (0.0%) | 4 (0.3%) | 5 (0.1%) |
|  | Smoking status, n (%) |  |  |  |
|  | Never | 2,679 (52.9%) | 654 (50.5%) | 3,333 (52.4%) |
|  | Past | 1,919 (37.9%) | 471 (36.4%) | 2,390 (37.6%) |
|  | Current | 463 (9.1%) | 170 (13.1%) | 633 (9.9%) |
|  | Missing | 6 (0.1%) | 0 (0%) | 6 (0.1%) |
|  | Physical activity, n (%) |  |  |  |
|  | Rarely/never | 2,155 (42.5%) | 586 (45.3%) | 2,741 (43.1%) |
|  | <1 time/week | 844 (16.7%) | 195 (15.1%) | 1,039 (16.3%) |
|  | 2-3 times/week | 1,470 (29.0%) | 349 (26.9%) | 1,819 (28.6%) |
|  | >=4 times/week | 593 (11.7%) | 165 (12.7%) | 758 (11.9%) |
|  | Missing | 5 (0.1%) | 0 (0%) | 5 (0.1%) |
|  | Blood Pressure, n (%) |  |  |  |
|  | <140/90 mm Hg | 3,072 (60.6%) | 741 (57.2%) | 3,813 (59.9%) |
|  | >=140/90 mm Hg | 1,994 (39.4%) | 553 (42.7%) | 2,547 (40.0%) |
|  | Missing | 1 (0.0%) | 1 (0.1%) | 2 (0.0%) |
|  | Healthy Diet*, n (%) |  |  |  |
|  | Healthy | 745 (14.7%) | 171 (13.2%) | 916 (14.4%) |
|  | Not healthy | 4,151 (81.9%) | 1,068 (82.5%) | 5,219 (82.0%) |
|  | Missing | 171 (3.4%) | 56 (4.3%) | 227 (3.6%) |
|  | History of Diabetes, n (%) |  |  |  |
|  | No | 4,903 (96.8%) | 1,235 (95.4%) | 6,138 (96.5%) |
|  | Yes | 163 (3.2%) | 60 (4.6%) | 223 (3.5%) |
|  | Missing | 1 (0.0%) | 0 (0%) | 1 (0.0%) |
|  | Total Cholesterol, n (%) |  |  |  |
|  | <200mg/dL | 1,403 (27.7%) | 332 (25.6%) | 1,735 (27.3%) |
|  | >200mg/dL | 3,465 (68.4%) | 882 (68.1%) | 4,347 (68.3%) |
|  | Missing | 199 (3.9%) | 81 (6.3%) | 280 (4.4%) |
|  | Life's Simple 7 Score, n (%) |  |  |  |
|  | Poor (0-3) | 1,549 (30.6%) | 397 (30.7%) | 1,946 (30.6%) |
|  | Intermediate (4-5) | 2,578 (50.9%) | 615 (47.5%) | 3,193 (50.2%) |
|  | Optimal (6-7) | 535 (10.6%) | 139 (10.7%) | 674 (10.6%) |
|  | Missing | 405 (8.0%) | 144 (11.1%) | 549 (8.6%) |

Min = Minimum, Max = Maximum, SD = Standard Deviation

*Healthy diet was defined as meeting at least two of the following: ≥4.5 cups/day of fruits and vegetables, ≥2 servings/week of fish, ≥3 servings/day of fiber-rich whole grains, <1500 mg/day of sodium, and <36 oz/week of sugar-sweetened beverages

**Supplementary Table 2:** Cognitive test performance at each cognitive assessment by Stroke Belt residence at baseline (*N* = 6,362)

|  | **Living outside the Stroke Belt**  (*n* = 5,231) | **Living inside the Stroke Belt**  (*n* = 1,131) | **Overall**  (*N* = 6,362) |
| --- | --- | --- | --- |
| **Immediate East Boston Memory Test** |  |  |  |
| **1^st^ assessment** |  |  |  |
| Mean (SD) | 9.58 (1.59) | 9.58 (1.58) | 9.58 (1.59) |
| Median [Min, Max] | 10.0 [0, 12.0] | 10.0 [3.00, 12.0] | 10.0 [0, 12.0] |
| Missing | 5 (0.1%) | 0 (0%) | 5 (0.1%) |
| **2^nd^ assessment** |  |  |  |
| Mean (SD) | 9.66 (1.70) | 9.69 (1.71) | 9.66 (1.70) |
| Median [Min, Max] | 10.0 [0, 12.0] | 10.0 [0, 12.0] | 10.0 [0, 12.0] |
| Missing | 568 (10.9%) | 111 (9.8%) | 679 (10.7%) |
| **3^rd^ assessment** |  |  |  |
| Mean (SD) | 9.65 (1.76) | 9.63 (1.82) | 9.64 (1.77) |
| Median [Min, Max] | 10.0 [0, 12.0] | 10.0 [2.00, 12.0] | 10.0 [0, 12.0] |
| Missing | 929 (17.8%) | 215 (19.0%) | 1144 (18.0%) |
| **Delayed East Boston Memory Test** |  |  |  |
| **1^st^ assessment** |  |  |  |
| Mean (SD) | 9.28 (1.79) | 9.27 (1.77) | 9.28 (1.79) |
| Median [Min, Max] | 10.0 [0, 12.0] | 10.0 [0, 12.0] | 10.0 [0, 12.0] |
| Missing | 13 (0.2%) | 3 (0.3%) | 16 (0.3%) |
| **2^nd^ assessment** |  |  |  |
| Mean (SD) | 9.35 (1.93) | 9.39 (2.01) | 9.35 (1.95) |
| Median [Min, Max] | 10.0 [0, 12.0] | 10.0 [0, 12.0] | 10.0 [0, 12.0] |
| Missing | 573 (11.0%) | 111 (9.8%) | 684 (10.8%) |
| **3^rd^ assessment** |  |  |  |
| Mean (SD) | 9.29 (2.05) | 9.32 (2.03) | 9.30 (2.05) |
| Median [Min, Max] | 10.0 [0, 12.0] | 10.0 [0, 12.0] | 10.0 [0, 12.0] |
| Missing | 933 (17.8%) | 216 (19.1%) | 1149 (18.1%) |
|  |  |  |  |
| **10 Word List  Telephone Interview for Cognitive Status** |  |  |  |
| **1^st^ assessment** |  |  |  |
| Mean (SD) | 3.02 (2.10) | 2.81 (2.11) | 2.98 (2.10) |
| Median [Min, Max] | 3.00 [0, 10.0] | 3.00 [0, 10.0] | 3.00 [0, 10.0] |
| Missing | 13 (0.2%) | 3 (0.3%) | 16 (0.3%) |
| **2^nd^ assessment** |  |  |  |
| Mean (SD) | 3.49 (2.30) | 3.42 (2.29) | 3.48 (2.30) |
| Median [Min, Max] | 3.00 [0, 10.0] | 3.00 [0, 10.0] | 3.00 [0, 10.0] |
| Missing | 573 (11.0%) | 111 (9.8%) | 684 (10.8%) |
| **3^rd^ assessment** |  |  |  |
| Mean (SD) | 3.46 (2.27) | 3.23 (2.25) | 3.42 (2.27) |
| Median [Min, Max] | 3.00 [0, 10.0] | 3.00 [0, 10.0] | 3.00 [0, 10.0] |
| Missing | 937 (17.9%) | 216 (19.1%) | 1153 (18.1%) |
|  |  |  |  |
| **Animal Naming Test** |  |  |  |
| **1^st^ assessment** |  |  |  |
| Mean (SD) | 17.6 (4.95) | 17.0 (4.94) | 17.5 (4.95) |
| Median [Min, Max] | 17.0 [0, 43.0] | 17.0 [5.00, 34.0] | 17.0 [0, 43.0] |
| Missing | 21 (0.4%) | 3 (0.3%) | 24 (0.4%) |
| **2^nd^ assessment** |  |  |  |
| Mean (SD) | 18.3 (5.28) | 17.5 (5.21) | 18.2 (5.27) |
| Median [Min, Max] | 18.0 [0, 49.0] | 17.0 [4.00, 46.0] | 18.0 [0, 49.0] |
| Missing | 572 (10.9%) | 111 (9.8%) | 683 (10.7%) |
| **3^rd^ assessment** |  |  |  |
| Mean (SD) | 17.9 (5.13) | 17.2 (5.30) | 17.8 (5.17) |
| Median [Min, Max] | 18.0 [4.00, 49.0] | 17.0 [5.00, 40.0] | 17.0 [4.00, 49.0] |
| Missing | 930 (17.8%) | 215 (19.0%) | 1145 (18.0%) |
|  |  |  |  |
| **Telephone Interview for Cognitive Status** |  |  |  |
| **1^st^ assessment** |  |  |  |
| Mean (SD) | 34.3 (2.73) | 34.0 (2.70) | 34.2 (2.73) |
| Median [Min, Max] | 34.0 [4.00, 41.0] | 34.0 [16.0, 41.0] | 34.0 [4.00, 41.0] |
| Missing | 12 (0.2%) | 3 (0.3%) | 15 (0.2%) |
| **2^nd^ assessment** |  |  |  |
| Mean (SD) | 34.2 (2.85) | 33.9 (3.04) | 34.2 (2.89) |
| Median [Min, Max] | 34.0 [16.0, 41.0] | 34.0 [13.0, 41.0] | 34.0 [13.0, 41.0] |
| Missing | 572 (10.9%) | 111 (9.8%) | 683 (10.7%) |
| **3^rd^ assessment** |  |  |  |
| Mean (SD) | 34.3 (3.02) | 33.9 (2.98) | 34.2 (3.02) |
| Median [Min, Max] | 35.0 [12.0, 41.0] | 34.0 [22.0, 41.0] | 34.0 [12.0, 41.0] |
| Missing | 929 (17.8%) | 215 (19.0%) | 1144 (18.0%) |

**Supplementary information on vital status**

Given the information available in the dataset, we approximated mortality prior to the second and final assessments by identifying deaths occurring before the median dates of the recorded second and final interviews.
Following this approach the number of deaths before the second interview was *N* = 98 and *N* = 248 before the last cognitive assessment.
